# Cold in the Head

**Published:** 1882-08

**Authors:** 


### COLD IN THE HEAD.

When a person takes a cold it will "settle" in the head, throat, chest, bowels, or joints, according to circumstances; if in the head, inducing an unpleasant "stuffing up" and an interruption of the sense of smell. An immediate and grateful relief is experienced sometimes by applying a smelling-bottle (hartshorn) to the nose and keeping it there until it begins to be felt, then remove the bottle for a moment and reapply as before; this is repeated seven or eight times in the course of a few minutes—the nostrils are freed and the sense of smell restored. This same hartshorn gives almost instant relief from the effects of the poisonous bites of all insects, vermin and reptiles by bathing the parts bitten, very freely.

